# Production, Transmission, Pathogenesis, and Control of Dengue Virus: A Literature-Based Undivided Perspective

**DOI:** 10.1155/2021/4224816

**Published:** 2021-12-15

**Authors:** Muhammad Torequl Islam, Cristina Quispe, Jesús Herrera-Bravo, Chandan Sarkar, Rohit Sharma, Neha Garg, Larry Ibarra Fredes, Miquel Martorell, Mohammed M. Alshehri, Javad Sharifi-Rad, Sevgi Durna Daştan, Daniela Calina, Radi Alsafi, Saad Alghamdi, Gaber El-Saber Batiha, Natália Cruz-Martins

**Affiliations:** ^1^Department of Pharmacy, Life Science Faculty, Bangabandhu Sheikh Mujibur Rahman Science and Technology University, Gopalganj (Dhaka)8100, Bangladesh; ^2^Facultad de Ciencias de la Salud, Universidad Arturo Prat, Avda. Arturo Prat 2120, Iquique 1110939, Chile; ^3^Departamento de Ciencias Básicas, Facultad de Ciencias, Universidad Santo Tomas, Chile; ^4^Center of Molecular Biology and Pharmacogenetics, Scientific and Technological Bioresource Nucleus, Universidad de La Frontera, Temuco 4811230, Chile; ^5^Department of Rasa Shastra & Bhaishajya Kalpana, Faculty of Ayurveda, Institute of Medical Sciences, Banaras Hindu University, Varanasi-221005, Uttar Pradesh, India; ^6^Department of Medicinal Chemistry, Institute of Medical Sciences, Banaras Hindu University, Varanasi-221005, Uttar Pradesh, India; ^7^European Institute of Traditional Chinese Studies, 4000-501 Porto, Portugal; ^8^Department of Nutrition and Dietetics, Faculty of Pharmacy, and Centre for Healthy Living, University of Concepción, 4070386 Concepción, Chile; ^9^Universidad de Concepción, Unidad de Desarrollo Tecnológico, UDT, Concepción 4070386, Chile; ^10^Pharmaceutical Care Department, Ministry of National Guard-Health Affairs, Riyadh, Saudi Arabia; ^11^Facultad de Medicina, Universidad del Azuay, Cuenca, Ecuador; ^12^Department of Biology, Faculty of Science, Sivas Cumhuriyet University, 58140 Sivas, Turkey; ^13^Beekeeping Development Application and Research Center, Sivas Cumhuriyet University, 58140 Sivas, Turkey; ^14^Department of Clinical Pharmacy, University of Medicine and Pharmacy of Craiova, 200349 Craiova, Romania; ^15^Laboratory Medicine Department, Faculty of Applied Medical Sciences, Umm Al-Qura University, Makkah, Saudi Arabia; ^16^Department of Pharmacology and Therapeutics, Faculty of Veterinary Medicine, Damanhour University, Damanhour, Egypt; ^17^Faculty of Medicine, University of Porto, Porto, Portugal; ^18^Institute for Research and Innovation in Health (i3S), University of Porto, Porto, Portugal; ^19^Institute of Research and Advanced Training in Health Sciences and Technologies (CESPU), Rua Central de Gandra, 1317, 4585-116 Gandra PRD, Portugal

## Abstract

Dengue remains one of the most serious and widespread mosquito-borne viral infections in human beings, with serious health problems or even death. About 50 to 100 million people are newly infected annually, with almost 2.5 billion people living at risk and resulting in 20,000 deaths. Dengue virus infection is especially transmitted through bites of *Aedes* mosquitos, hugely spread in tropical and subtropical environments, mostly found in urban and semiurban areas. Unfortunately, there is no particular therapeutic approach, but prevention, adequate consciousness, detection at earlier stage of viral infection, and appropriate medical care can lower the fatality rates. This review offers a comprehensive view of production, transmission, pathogenesis, and control measures of the dengue virus and its vectors.

## 1. Introduction

Dengue regards one of the utmost serious arboviral infections around the world. dengue virus (DENV) is transmitted through bites of female *Aedes* mosquitos especially *Aedes (Stegomyia) aegypti*, *Ae. alpopictus* [1], *Ae. niveus*, and *Ae. polynesiensis* [2]. DENV infection is almost similar to flu-like infection and sometimes develops into possibly lethal difficulties or severe illness including dengue shock syndrome and dengue hemorrhagic fever. World Health Organization (WHO) reports that DENV infection has been shown to 30-fold increase around the globe over the past five decades, and approximately 100 million newly infected people are estimated in over 100 endemic countries with 20,000 deaths annually [3].

DENV and its vectors are primarily noticed in tropical and subtropical environments globally, frequently in urban and semiurban areas. In Bangladesh, DENV has been detected as a severe health hazard. Between 2000 and 2008, 50,148 people were hospitalized for dengue in Bangladesh. But in August 2019, nearly 60,000 dengue patients have been hospitalized, and approximately 100 deaths have been reported. Severe DENV infection is a leading cause of tenacious sickness and deaths of people of all ages in Asian and Latin American countries. Unfortunately, we have no particular treatment strategy for dengue infection. It may be because historically our pharmaceutical section did not come up with much attention to this vector-borne viral disease.

This review is aimed at sketching a current scenario on DENV, dengue infection, and dengue vectors along with the production, transmission, pathogenesis, and ways of control of DENV, and its vectors offers a comprehensive view of production, transmission, pathogenesis, and control measures of DENV and its vectors.

## 2. Dengue Virus (DENV)

DENV, a pathogenic arthropod-borne flavivirus (arbovirus), is a single-stranded and positive-sense RNA molecule belonging to the family Flaviviridae.

The Flaviviridae family includes viruses transmitted by arthropods that cause serious illness in humans. The family includes a single genus-Flavivirus, with several types [4]. Recently, another subdivision of the family into three genera has been proposed as follows: genus Flavivirus includes arboviruses (yellow fever virus, dengue fever virus); genus Pestivirus-viruses involved in animal pathology; and genus Hepacavirus-the proposed name for different variants of hepatitis C virus [4].

**Figure 1:**
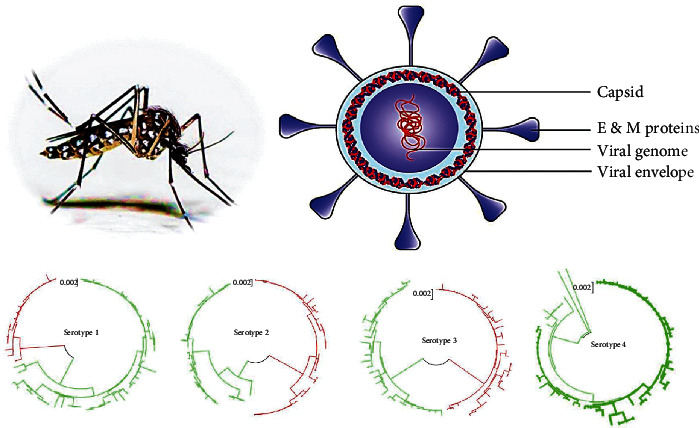
Diagram with dengue virus and its four serotypes.

To date, 47 strains of DENV have been identified. The total number of four closely linked serotypes (from DENV -1 to -4) of DENV has been identified to date, but they are lightly antigenically distinct [5, 6], and those can be subdivided into several genotypes according to their gene sequences [7]. These serotypes are generally progressed from a mutual ancestor, and all are considered as the causative agent of approximately similar disease spectrum in humans due to DENV selecting different receptors based on cell types and virus strains [8]. Developed viral particles have a spherical shape with 11 kb in length and 40-50 nm in diameter, containing single-stranded and positive-sense RNA molecule, which has a 5-methyl cap with a single open reading frame [2]. Dengue virus and its common four serotypes have shown in Figure 1.

### 2.1. DENV Vectors

**Figure 2:**
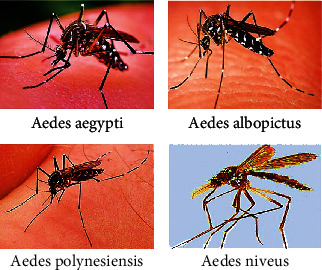
*Aedes* mosquitoes (dengue virus vectors).

Dengue virus infection usually spreads through bites of infected female mosquitos of genus *Aedes*, especially by the *Aedes aegypti* and *Ae. albopictus* [1]. However, the other two vectors such as *Ae. polynesiensis* and *Ae. niveus* have been identified as the secondary vectors in some regions throughout the world [2] (Figure 2).

Adult *Ae. aegypti* has a white scale that forms a lyre or violin shape at the dorsal side of the thorax, while the adult *Ae. albopictus* forms a white stripe at the middle point of the top of the thorax region. The white bands of every tarsal segment of the hind legs of these mosquitos are known as the white stripe. The abdomen is generally found to be black or dark brown, but sometimes, it also bears white scales. Females are usually larger than males; on the other way, through finding small palps tipped with silver or white scales, they can be discriminated against properly. Males are specially identified by the plumose type of antennae. On the other hand, females are seen to bear short hair. Under a microscope, the mouthparts of the male are watched as a structural modification for nectar feeding, and female mouthparts are viewed as a modified structure for feeding on blood. The darkly coloured proboscis is found to be present in both sexes. In addition, two clusters of white scales presented on the segment above the proboscis are known as clypeus. The tip of the abdomen is pointed out as a distinctive feature of all *Aedes* species [9].

### 2.2. Geographical Distribution

DENV mainly originated from monkeys, then jumped to humans in Southeast Asia or Africa between 100 and 800 years ago. Geographically DENV has been restricted till the 1950s, but after the Second World War caused a rapid distribution throughout the world. Firstly, DENV infection was recognized in the Thailand and Philippines in the 1850s, and after the 1980s towards Latin America and the Caribbean. Presently, DENV is prevalent throughout the different countries (at least 100 countries) including in Asia, the Pacific, the Americas, Africa, and the Caribbean. DENV epidemics occurred in 26 states [10]. Scientific reports demonstrate that DENV-2 and 3 serotypes were mostly outbroken before 2000 and between 2000 and 2009, respectively. DENV-1 serotype started to dominate worldwide dengue outbreaks and after 2010, the DENV-4 [11].

**Figure 3:**
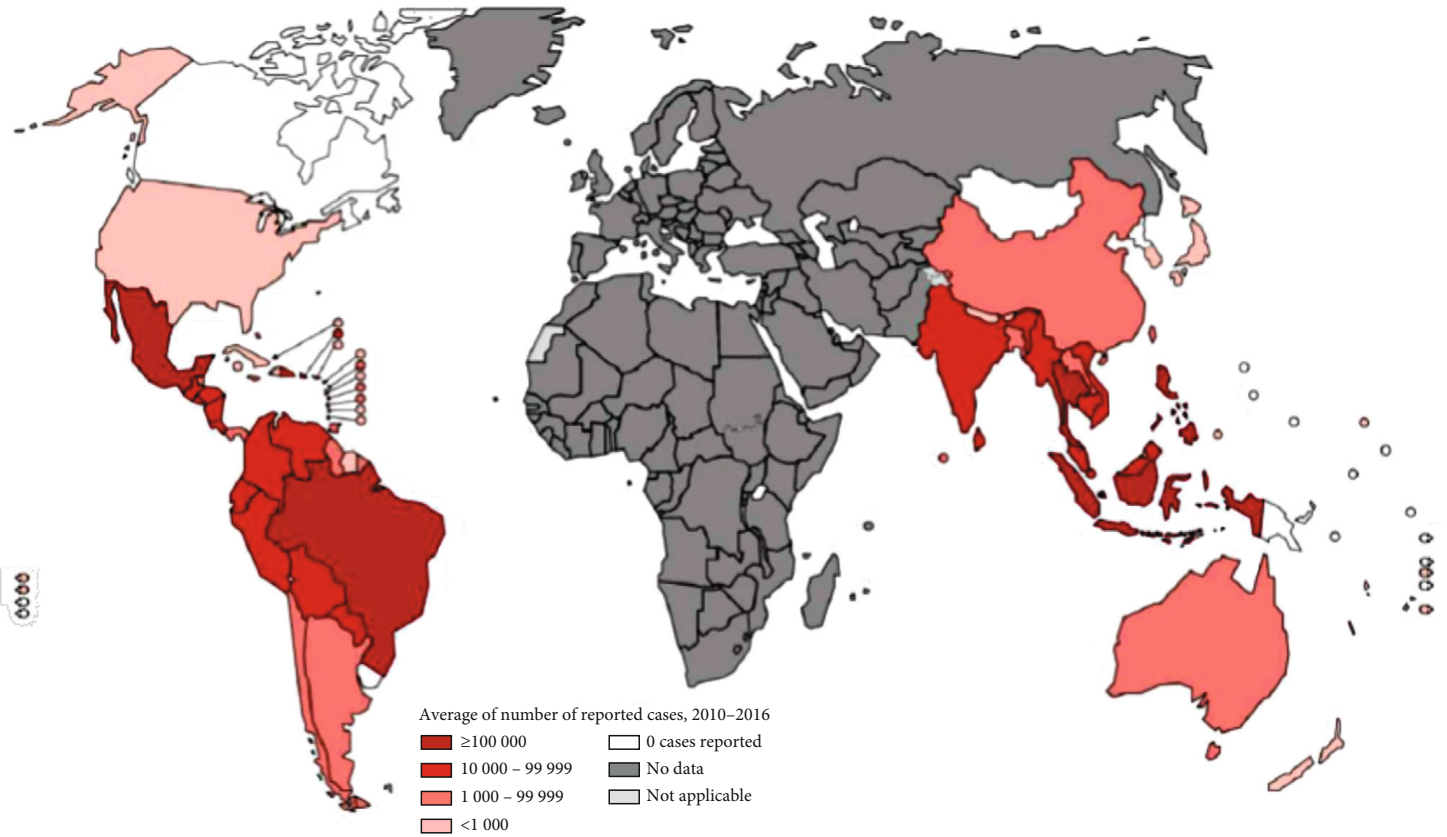
Geographical distribution of dengue worldwide.

The geographical distribution of DENV worldwide has been shown in Figure 3.

*Ae. Aegypti* is scattered in tropical areas geographically, and it breeds artificially in containers (such as tyres, drums, flower vases including plastic food containers, tin cans, and old motor parts) that are filled with water [12].

*Ae. aegypti* is an insect of holometabolous type, which is fully developed after completing metamorphosis (i.g., four growing phases from egg to adult period). The duration of the life span of an adult may be 2 to 4 weeks; however, it depends on the environmental conditions, at least 4-5 times a female mosquito lays eggs throughout her whole life span and the average 10 to 100 eggs in a single spawn. Three diverse polytypic forms are found in *Ae. aegypti* such as sylvan, domestic, and peridomestic [13].

A sylvan type is generally a rural form which breeds in tree holes, normally in forests; the domestic type commonly breeds in municipal surroundings, frequently inside or around houses; and the peridomestic type usually survives in biologically modified regions as groves and coconut farms [14]. *Ae. aegypti* can survive above 4°C [15]; on the other hand, about 15-37°C temperature is required for a complete life cycle [16].

The extent of DENV epidemics not only depends on the presence of DENV and mosquito genotypes but also depends on how they interrelate with local temperature [17]. Nevertheless, a current study demonstrated that DENV infection can alter gene expression in the *Ae. aegypti* mosquito's head that causes a loss of their olfactory preferences, thereby modifying oviposition site choice [18]. Now, the question is how safe is the host nervous system's homeostasis during Dengue infection?

### 2.3. Life Cycle

**Figure 4:**
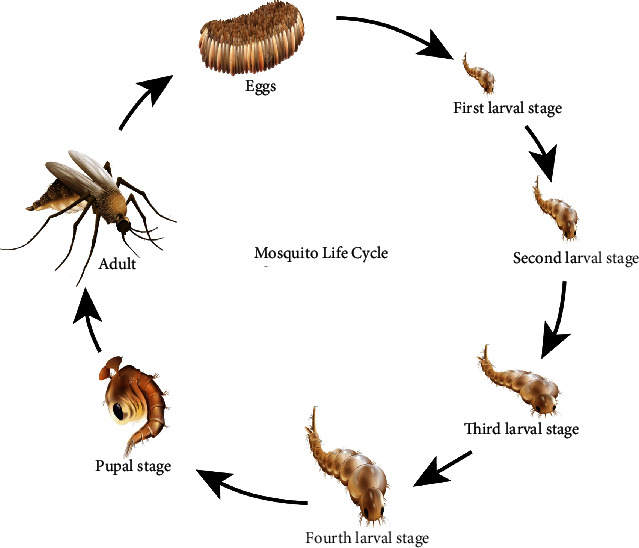
Mosquito life cycle.

Primarily, the DENV was transmitted via sylvatic cycles in Asia and Africa by *Aedes* mosquito and the nonhuman primates, with occasional appearances of human populations [19]. However, nowadays, the global spread of DENV follows its emergence of all types of transmissions (e.g., sylvatic cycles and vertical: mosquito to mosquito). Thus, its primary life cycle entirely involves the transmission between *Aedes* mosquitoes and humans [20]. One report suggests that dogs or other animals may act as incidental hosts and may serve as reservoirs of DENV infection [21]. Life cycles of mosquitoes have been shown in Figure 4.

### 2.4. Immune Defensive Pathways

The Toll pathway is one of the well-known potential immune defensive pathways against the DENV and its serotypes bearing *Ae. aegypti* [22]. In a study, after ten days postinfection of DENV, the antioxidant enzymes were found to suppress, while upregulated the expressions of Toll, JAK-STAT, and pathogen recognition receptor (PRR) [23]. It has also been analyzed that the JAK-STAT pathway is another important DENV defensive pathway in invertebrates [24, 25]. The mosquitoes of genus *Aedes* should be more vulnerable to DENV infection if the receptor JAK homolog HOP or Dome is inhibited by RNA inference (RNAi, e.g., ds RNA and prM RNA) [25, 26].

In a study, miR-375 was found to enhance DENV2 replication capacity [27]. In another study, in the period of DENV infection, miRNAs were identified in different forms (about sixty-six) in *Ae. albopictus*, where upregulated miR-34-5p targets the Toll pathway signalling protein (REL-1) [28]. Conversely, downregulated peptidoglycan recognition protein LE, and AMP defensin D. miR87 targets the Toll pathway [28].

Differential expression of miRNAs in DENV has been also reported by Yen et al. [29]. In this study, the authors highlighted the possibility of using artificial antiviral miRNAs to reduce the transmission of two major arboviruses in transgenic Ae. Aegypti. The miRNA-based approach resulted in a dual resistance phenotype for Dengue serotype 3 viruses (DENV-3).

The piRNAs also plays essential roles in the innate antiviral response in DENV [30–33]. Moreover, nonretroviral integrated RNA viruses (NIRVS) were recognized in *Ae. aegypti* and *Ae. albopictus* in a larger number [34].

The expression of cecropin-like AMPs was expressively upregulated by the infection of DENV [24]. In *Ae. aegypti*, the immune deficiency (IMD) pathway shows a significant role to resist DENV susceptibility, while the increase in viral replication [35]. The ubiquitin variant (Ub3881) residues may inhibit the DENV envelope protein, thereby and decrease the production rate of DENV in *Aedes* vectors [36, 37].

The DENV-containing blood meal first appears in the midgut of the vectors, which has the first line of defense systems, such as the infection barrier and the escape barriers [38, 39]. It is evident that, after a successful entry of DENV, something has happened inside the midgut cells such as uncoating, replication, and new virus particle assembly. The innate immune signalling pathways have been seen to be effective during the infection of DENV in *Ae. aegypti*. Exogenous siRNA pathways also play a substantial role against DENV infections in the *Aedes* midgut [40]. DENV infection causes the production of NO in the hemolymph, where the virus is released into the hemocoel from the midgut. The hemocytes allow replication other than the distribution of DENV. Interestingly, DENV replication in hemocytes is extensively inhibited by NO [41]. It has been reported that about 40 differentially bacterial types have been isolated from the gut of *Ae. aegypti* through a gut microbiome study [42]. In another study, colonization of Csp_P in the midgut of the *Ae. aegypti* also inhibited DENV infection [43]. The *Talaromyces* (*Tsp*) secretome shows a considerable modulating effect on the midgut transcriptome. *Tsp* secretome may display a significant role in the advancement of DENV infection in the midgut through downregulating trypsin encoding genes involved in the digestion of blood and through reducing the enzymatic activity of trypsin [44]. It is cited that the presence of gram-negative endosymbiotic bacteria *Wolbachia* spp. in *Aedes* mosquitoes effectively suppressed the DENV infection [45]. *Wolbachia* activates antimicrobial peptides defensin and cecropin Toll pathway through producing reactive oxygen species (ROS) after inducing a reduction-oxidation (Redox) reaction in the mosquitos [46]. *Wolbachia* also upsurges vago1 expression in *Ae. aegypti* by acting as a ligand of the JAK-STAT pathway [47].

*Ae. aegypti* macroglobulin complement related factor (AaMCR) recognizes DENV particles. An anti-DENV effect on *Aedes* mosquitoes has been found to link with the upregulation of AMP expression in the hemocytes [48]. The salivary glands also contain the infection barrier and the escape barriers [49]. Moreover, incomplete apoptosis of DENV occurs here, which is required for the virus to release via saliva [50]. A study revealed that multiple immune defensive pathways (e.g., Toll and IMD) can be found here, and this can rise putative antibacterial proteins/peptides (e.g., attacin, cecropins, defensins,and gambicin) [24, 28, 35, 48, 51]. In the brain of *Ae. aegypti*, a homolog of *Hikaru Genki* (*AaHig*) has been found to express ubiquitously [52].

Lipid droplets (LDs) containing a few exclusive structural proteins (Perilipin 1, 2, and 3) and a fatty acid monolayer are exclusively found to present in a variety of organisms including DENV. These have been found to provide immunological defense of *Aedes* mosquitoes [53–55].

## 3. DENV Infection

### 3.1. Transmission

After initial midgut infection, DENV distributes systemically through the body cavity (commonly known as hoemocel) of *Aedes* vectors, after that way disseminates in secondary tissues. The time taken between initial midgut infection and successive transmission of DENV by its vector (e.g., *Ae. aegypti*) is termed as extrinsic incubation period (7 to 14 days at 25-30°C). DENV stays in the midgut of the vectors which it may be due to the viral genome being stable here [36].

The ubiquitin-proteasome, an important pathway, acts significant activity in the regulation of infectious DENV production in vectors [36]. Finally, an infection of the salivary glands and the release of virions into the host's saliva occur throughout the DENV transmission to the host [56]. Blood cells and plasma are important media for the four serotypes of DENV spreading into the host. A relation of domain III from the envelope glycoprotein of DENV-II with human plasma proteins has been identified by Huerta et al. [57] [57]. DENV infection inductees after the attachment of the dengue virus to the target cell through interfaces between the various cell surface receptors and viral envelope (E) protein [58]. In mammalian cells, all categorized serotypes interact with mannose, heparan sulfate, nLc4Cer, and DC-SIGN/L-SIGN receptors.

Additionally, the DENV-2 serotype is found to intensity of binding with GRP78, CD14-associated protein, HSP70/HSP90, and two other unidentified receptor proteins. Conversely, serotypes DENV 1-3 bind with the laminin receptor while serotypes DENV 2-4 attach with an unknown protein receptor [59].

DENV after receptor-mediated endocytosis, virion fuses with acidic lysosomes, and its genomic RNA is released into the cytoplasm and translated into a polyprotein of ~3400 amino acids (genome is about 11000 bases of positive-sense, a single-stranded RNA (ssRNA)) that are further cleaved by viral and host proteases into three structural (capsid: C, membrane: M, and envelope: E) and seven nonstructural (NS1, NS2A, NS2B, NS3, NS4A, NS4B and NS5) proteins [60].

C protein is a foremost structural component of DENV that is localized in the cytoplasm and nuclei [61, 62]. The nuclear localization of this protein is thought to be crucial for its well-organized replication [61, 63].

The lipid bilayer of virions is formed by lipid (approximately 17% by weight) between the nucleocapsid core and E/M outer shell [64, 65]. During the replication of DENV, a membrane-bound replication complex formation helps to incorporate host factors, viral proteins, and genomic RNA [66]. In this case, positive-strand (+) DENV genomic RNA acts as a template to synthesize complementary negative-strand (-) RNA, which is sequentially used for multiple (+) RNA genomes production that is obtainable for translation and regulation of replication cycles or packaging into virions [67]. However, in a study, Raquin and Lambrechts [68] showed the presence of DENV genomic RNA in the salivary galnds of *Ae. aegypti*, indicating an active replication of DENV in its vector prior to transmission [68]. DENV itself encodes RNA-dependent RNA polymerases, and the infection cycle of this virus is catalyzed by other cellular factors [69].

DENV infections can alter many important proteins in the subcellular locales, including the Alix apoptosis-linked gene-2-interacting protein X; therefore, blocking this step may be one of the innovative beneficial approaches to reduce DENV replication in the host [70]. Instead, several genes have been identified that reduce infection of DENV when silenced by at least 60% in its most important vector *Ae. aegypti*. Among them, a putative cysteine-rich venom protein SeqID AAEL000379 (CRVP379) silencing has been found to reduce DENV infection significantly in the cells of midgut tissues of *Ae. aegypti* [71].

Loqs2, a gene has been found only in *Aedes* mosquitoes, which is essential for the appropriate effectiveness of RNA interference in this type of mosquito. However, without Loqs2, the viruses can multiply and consequently infect their salivary glands [72].

An interaction between DENV nonstructural protein 4A (NS4A) and host cellular vimentin has been demonstrated in localizing and concentrating the viral replication complex at the perinuclear site, in consequence assisting well-organized replication of viral RNA [73].

**Figure 5:**
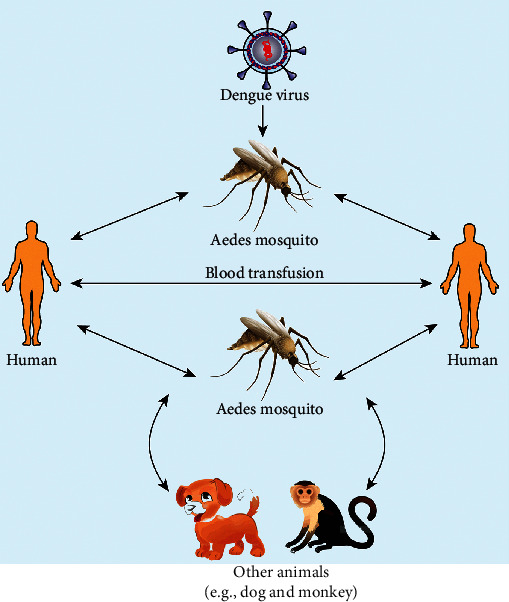
DENV transmission mode between the vector and hosts.

Figure 5 shows a general DENV transmission mode in its vector to hosts.

### 3.2. Pathogenesis

DENV is usually greater in tropical and subtropical environments throughout the world, frequently in urban and semiurban zones. People, exposed to infected mosquitoes of all ages are susceptible to DENV infection. DENV infection causes dandy fever, breakbone fever, and dengue hemorrhagic fever; and in severe cases, dengue shock syndrome has occurred. The rainy season is the most favorable climate for DENV infection outbreaks in tropical countries in Asia and South America. Generally, the infected female *Aedes* mosquitoes transmit DENV in humans. Although humans are not capable of transmitting DENV, it can be transmitted during the blood transfusion between an infected person to a noninfected (healthy) person [74, 75].

### 3.3. Physiological Data

After the incubation period (3 to 14 days) of DENV, the person may experience one or more early symptoms such as nausea, headache, rash, fever, musculoskeletal pain, and joint pain [76]. In classic dengue fever, body temperature ranges from 39 to 40°F (5-7 days) [77]. In the meantime, the DENV may enter systematically into the bloodstream at the peripheral zones and sequentially damage lymph nodes and blood vessels resulting in dengue hemorrhagic fever [78]. Symptoms of the latter case include bleeding under the skin and from the gums and nose. On the other hand, difficulty in breathing appears in patients having dengue hemorrhagic fever, and severe progress of it can lead to dengue shock syndrome, if left untreated, can result in death.

### 3.4. Micronutrient Imbalance

The morphogenesis and translation and/or replication of DENV occur in the endoplasmic reticulum (ER) [79], where Ca^2+^ plays a significant activity in cell signaling. The immune response of T-cell has been drawn in DENV infection. At the time of secondary infection (i.e., infection after 1-2 days of fever onset), high concentration of interferon-alpha (IFN-*α*) is found, while high levels of soluble interferon *γ* (IFN-*γ*), interleukin 2 receptor (IL-2R), and soluble CD4 and CD8 were reported throughout the outset of vascular permeability [80, 81]. Dengue antigen is evident to increase the influx of Ca^2+^ into T-cells, thus reducing blood Ca^2+^ levels [82, 83].

A multifunctional intermediate messenger protein calmodulin is well known as a primary sensor of intracellular Ca2+ in the eukaryotic cells, which plays imperative utility for proper decoding of Ca2+ signalling [84]. DDX3X is a DEAD-box RNA helicase, which binds with the TRPV4 cation channel that regulates its functions. DDX3X is released by the TRPV4-mediated Ca^2+^ influx; at the same time, DDX3X nuclear translocation is derived through a process involving calmodulin and its kinase II-dependent pathway.

Therefore, pharmacological inhibition or genetic depletion of TRPV4 can diminish DDX3X-dependent functions, including translation and nuclear viral export. Thus, targeting TRPV4 may reduce the infectivity of some viruses, including dengue, Zika viruses, and hepatitis C [85]. In a study, the effect of W-7, a calmodulin antagonist in DENV infection in Huh-7 cells, was seen, where W7 was inhibited viral yield, NS1 secretion and viral RNA, and protein synthesis, possibly through direct inhibition of NS2B-NS3 activity and/or inhibition of the interaction between NS2A with Ca^2+^-calmodulin complex [86]. Calcium depletion can modulate cardiac functions, immunopathogenesis, and platelet functions in dengue infection [82]. Another study on 36 h postinfection of Huh7 cells has been demonstrated that calcium modulating cyclophilin-binding ligand influences the apoptosis process by changing the activation of caspase-3 and the potentiation of mitochondrial membrane [87].

### 3.5. Clinical Aspects

In most cases, asymptomatic or mildly symptomatic pathways are promising ways for transiting DENV infection [88].

The most common signs and symptoms include pain of bone, joint, muscle, and retro-orbital; headache; fever (40°C); maculopapular or macular rash; and minor hemorrhagic manifestations including purpura, malaise, ecchymosis, petechiae, epistaxis, hematuria, bleeding gums, aches or pain, or a positive tourniquet test result. Dengue fever lasts from 3 to 7 days. Before appearing the warning signs of severe DENV infection, a slight portion of the infected patients goes to life-threatening conditions [89].

Severe DENV infection can cause organ impairment, bleeding, and plasma leakage. The warning signs during dengue infection include vomiting, abdominal pain, respiratory distress, clinical/fluid accumulation, lethargic condition, mucosal bleeding, liver enlargement (>2 cm), restlessness, lethargic condition, and rapid decrease in platelet count. An intensive care should be taken for the patients having infancy, pregnancy, chronic hemolytic diseases, renal failure, diabetes, obesity, and old age [2]. Chronic infections of DENV may preserve in the central nervous system and can be considered in progressive dementia patients [90].

In a recent study by Suppiah et al., the link between clinical manifestation characteristic of Dengue fever and genotypes, respectively, and DENV-specific phenotypes, was highlighted. Thus, it was found that the clinical symptoms are more severe in patients infected with DENV 2 serotype, compared to patients infected with DENV1 serotype. Musculoskeletal manifestations are characteristic of DENV serotype 3 infection [91]. Also, nonstructural proteins (e.g., NS1, NS3, and NS5) can be targeted to develop a novel vaccine strategy [92, 93].

### 3.6. Diagnosis

Unfortunately, still, the signs and symptoms are the foremost tools for the DENV infection diagnosis [94, 95]. Fever or flu-like fever is the initial tool of DENV infection.

To date, the well-known tests for detecting the presence of DENV include identification of the responsible viral genomic sequences, DENV serotype, viral antigen(s) (e.g., NS1 by MAC-ELISA assays) and/or antibodies in response to it (e.g., IgG, IgM), and platelet counts.

Other important diagnosis includes viral RNA detection (by nucleic acid amplification tests (NAAT) or RT-PCR), detection of dengue specific monoclonal antibodies, IgM captured ELISA, alive and/or viral isolation from mosquito cell lines [96–100]. Immune-fluorescence tests, capture ELISA, and hemagglutination assays are the commonly used laboratory methods [101]. Other test includes +ve tourniquet test, leukopenia, HCT concurrent with a rapid decrease in platelet count, AST or ALT ≥ 1000 IU/L, and impaired consciousness [102]. Some important diagnostic approaches and methodology have been shown in Table 1.

## 4. Control of DENV and Its Vectors

Public awareness counts as one of the major consequences of the management of DENV, which essentially helps to avoid or inhibit the contacts of the infected *Aedes* mosquitoes or other animals and their derivatives [103]. In this way, *Ae. aegypti* was properly eradicated during the 1960s from different areas of the USA. For this, a well-educated society needs the strongest collaborative activities with skilful and well-trained mosquito control staff [104].

It is possible to control DENV infection by using different interesting methods.

### 4.1. Preventive Measures

Preventive measures should be the first and best choice in this case, such as the prevention of direct contact of blood or blood-derived products from the infected patients and infected vectors from the infected host [105]. Daytime is the most suitable time for biting *Aedes* mosquitoes; consequently, its contact can be diminished or avoided using the following techniques:

- By using nets (e.g., insecticide-treated nets) and mosquito repellents (e.g., coils, solids (sticks), aerosols, liquids, pump sprays, and nonsticky creams)
- By wearing gloves and other defensive clothing
- Through well-planed management of wastes and stored water
- By destroying the mature *Aedes* mosquitoes or larvae through applying some protective chemicals (e.g., N,N-Diethyl-3-Methylbenzamide, diethyl carbonate, metofluthrin, oil of lemon-eucalyptus, diethyl phthalate, ethyl hexanediol, and picaridin) [106, 107].

CYD-TDV (brand name Dengvaxia), an one and only FDA approved live-attenuated dengue vaccine prepared by applying rDNA technology through substituting the premembrane (PrM) and envelope (E) structural proteins of the 17D strain of attenuated yellow fever vaccine with those from the dengue serotypes excepting DENV-5 serotype, is manufactured by Sanofi Pasteur [108, 109]. Other vaccines under development are DENVax/TAK-003 (recombinant chimeric vaccine with DENV-1, -3, and -4 components on the DENV-2 backbone, developed at Mahidol University in Bangkok) [110, 111], TetraVax-DV (tetravalent admixture of monovalent vaccines, being tested in Brazil and Thailand in phase II trial) [112], TDEN PIV (inactivated tetravalent vaccine, being experimented by the Walter Reed Army Institute of Research and GSK in phase I clinical trials) [113], V180 (recombinant subunit vaccines expressed in Drosophila cells, undergoing phase I trial by Merck [114], and DNA vaccines (the Naval Medical Research Center attempted to develop a monovalent DNA plasmid vaccine) [111].

### 4.2. The Role of Natural Products and Their Bioactive Constituents in Controlling DENV Infection

Natural products are the potential sources of many important modern medicines [115–117]. Plants and/or their extracts having antidengue activities are also distributed worldwide [118–120]. To date, a number of medicinal plants have been reported to act against DENV and/or their vectors, for example, *Alternanthera philoxeroides* (Fam: Amaranthaceae) [121], *Azidarachta indica* (Fam: Meliaceae) [122], *Boesenbergia rotunda* (Fam: Zingiberaceae) [123], *Carica papaya* (Fam: Caricaceae) [124], *Cladosiphon okamuranus* (Fam: Chordariaceae) [78], *Cryptonemia crenulata* (Fam: Halymeniaceae) and *Gymnogongrus griffithsiae* (Fam: Phyllophoraceae) [125], *Cymbopogon citratus* (Fam: Poaceae), *Andrographis paniculata* (Fam: Acanthaceae), *Momordica charantia* (Fam: Cucurbitaceae), *Ocimum sanctum* (Fam: Labiatae), *Piper retrofractum* (Fam: Piperaceae) [126], *Flagellaria indica* (Fam: Flagellariaceae), *Cladogynos orientalis* (Fam: Euphorbiaceae), *Rhizophora apiculata* (Fam: Rhizophoraceae) and *Houttuynia cordata* (Fam: Saururaceae) [127], *Gymnogongrus torulosus* (Fam: Phyllophoraceae) [128], *Lippia alba* and *L. citriodora* (Fam: Verbenaceae) [129], *Meristiella gelidium* (Fam: Solieriaceae) [130], *Mimosa scabrella* (Fam: Fabaceae) [131], *Psidium guajava* (Fam: Myrtaceae) and *Euphorbia hirta* (Fam: Euphorbiaceae) (Abd [132]), *Quercus lusitanica* (Fam: Fagaceae) [133], *Tephrosia crassifolia*, *Tephrosia madrensis, Leucaena leucocephala*, and *Tephrosia viridiflora* (Fam: Fabaceae) [131, 134, 135], *Uncaria tomentosa* (Fam: Rubiaceae) [136], *Zostera marina* (Fam: Zosteraceae) [137], *Myristica fatua*, *Cymbopogon citratus* and *Acorus calamus* [138], *Doratoxylum apetalum* [139], *Psiloxylon mauritianum* [140], *Acorus calamus* (Fam: Acoraceae) [141], *Cinnamosma fragrans* [142], *Pedalium murex* [143], *Aesculus hippocastanum* [144], *Norantea brasiliensis* [145], *Azadirachta indica* [146], *Spondias mombin* [147], *Angelica sinensis* [148], *Phyllanthus* spp. [149], *Solanum xanthocarpum*, *Mesocyclops thermocyclopoides* (Mahesh [150]), *Delonix elata* (Fam: Fabaceae) [151], *Acalypha alnifolia* (Fam: Euphorbiaceae) [152], *Combretum collinum* [153], and *Solanum villosum* [154].

The aqueous extract of *Houttuynia cordata* (10-100 mg/mL) against DENV-2 with human hepatocarcinoma cell lineage (HepG2) cells showed that extract significantly decreased intracellular DENV-2 RNA production with the reduction in the expression of dengue protein. It also showed a potential role in the release of the virion from infected LLC-MK2 cells at 10-40 mg/mL concentrations [155]. 9 N-methylamine and Harmol may selectively inhibit DENV-2 multiplication without virucidal effect in cell cultures [156].

The ethyl acetate fraction of *H. cordata* and quercetin showed in vitro activity against mouse hepatitis virus (MHV) and DENV-2 with IC_50_ 0.98 and 125 *μ*g/mL for MHV while 7.50 and 176.76 *μ*g/mL for DENV-2 [157]. Delphinidin and epigallocatechin gallate showed a direct effect on against West Nile virus (WNV) and also reduced the infectivity of ZIKV and DENV. The effect of delphinidin and, particularly of epigallocatechin gallate, was found higher for the African strain (MR766) than for the American strain (PA259459) [158].

In another study, it was found an absence of anti-DENV activity in chemical constituents like acetyl-L-carnitine, melatonin, *α*-tocopherol, and folic acid while resveratrol exhibited some limited anti-DENV activity [159]. Organosulfur compounds in garlic were tested against DENV-2 NGC (New Guinea C) virus U937 human macrophage-like cells and Huh-7 human liver cells. The organosulfur compounds reduced the levels of proinflammatory cytokines (TNF-*α*, IL-8 and IL-10) and affect the oxidative stress response [160].

The methanol extract of *Rumex dentatus* showed the highest antiviral efficacy by inhibiting DENV-2 replication, with IC_50_ of 0.154 and 0.234 *μ*g/mL, while gallic acid showed with IC_50_ of 0.191 *μ*g/mL and 0.522 *μ*g/mL at 45 and 90 PFU of DENV-2 infection, respectively [161].

Naringenin (citrus flavanone) was evaluated against dengue viruses (serotypes 1–4) in Huh7.5 cells which impaired virus replication in human cells with IC_50_ = 35.81, 17.97, 117.1, and 177.5 *μ*M, respectively [162]. *Annona muricata* aqueous leave extract was evaluated against dengue virus type 2. Selectivity index of the extract was found more than 10 against DENV-2 which showed potential as a nature-based antiviral drug [163]. Three spirotetronate compounds (2EPS-A, -B, -C) isolated from *Actinomadura* strain showed strong DENV-2 NS2B-NS3 protease inhibition with IC_50_ values of 1.94 ± 0.18, 1.47 ± 0.15, and 2.51 ± 0.21 *μ*g/mL, respectively [164].

In vitro activity of essential oils of *β*-caryophyllene was evaluated against DENV-2. *β*-caryophyllene acts on the initial steps of the viral replication cycle and showed inhibition with IC_50_ = 22.5 ± 5.6 *μ*M against DENV-2 [165]. The ethanol extract of polyherbal formulation *Nilavembu kudineer showed* antiviral activity against DENV-2 virus infection in Vero and human macrophage cell line (THP-1 cells) from 0.78% till 0.01% of the human dose [166].

The aqueous leave extract of *Orthosiphon stamineus* was evaluated against DENV-2. The extract exhibited the ability to reduce DENV-2 replication in the pretreated cell while ineffective in inhibiting cell death in the posttreated cell [167]. Antiviral activity of natural alkaloid anisomycin was evaluated against DENV and ZIKV viruses. The compound prevented DENV and ZIKV multiplication in human cell lines, inhibited viral protein expression, and also impaired viral replication in the posttreated cell. In a lower dose, it also showed a significant decrease in viremia levels in ZIKV infected AG129 mice [168]. A natural antimicrobial agent (latarcin peptide) was evaluated against DENV replication in infected cells. The peptide exhibited a significant inhibitory effect (IC_50_ = 12.68 ± 3.2 *μ*M) against the dengue protease NS2B-NS3pro at room temperature and also reduced the viral RNA in a dose-dependent manner [169].

The crude extract of *Rhodiola imbricata* showed an antiviral immune response against the dengue virus. It induced interferon (IFN) b and other cytokines and also upregulated MIF-2a, PKR, and NF-*κ*B phosphorylation in infected cells [170]. The antiviral effect of *C. longa* extract showed low cytotoxicity and effective inhibitor (IC_50_ = 17.91 *μ*g/mL) against DENV-2 infected Huh7it-1 cells [171]. The hydroalcoholic extracts of leaves and bark of *Uncaria guinanensis* DENV-2-infected Huh-7 cells reduced intracellular viral antigen and inhibited the secretion of viral nonstructural protein [172]. The chemical compound 5-hydroxy-7-methoxy-6-methylflavanone inhibited DENV2 infectivity in LLC/MK2 (EC_50_ = 15.99 ± 5.38) as well as Vero cell lines (EC_50_ = 12.31 ± 1.64 *μ*M) and DENV4 (EC_50_ = 11.70 ± 6.04 *μ*M). Phospholipase A_2_, a chemical constituent of *Crotalus durissus terrificus* venom, was inhibited *dengue virus* and *yellow fever virus* infection in Vero cells, inducing a partial exposure of genomic RNA through glycerophospholipids cleavage [173]. Tomatidine has inhibited dengue virus mainly at late stages of infection towards all dengue virus serotypes and controlled the activating transcription factor 4 (ATF4) expression. It showed inhibition of DENV2 (EC_50_ = 0.82 *μ*M) infection mainly independent of ATF4 [174].

In silico analysis showed that Nimbin is found to be effective and reducing the morbidity and mortality against the envelope protein of DENV 1-4 infection [175]. Quinic acid derivatives were found effective against DENV1-4 and exhibited impaired dengue virus replication in infected Huh7.5 cell lines [176]. The antiviral activity of isobutyl gallate was evaluated against DENV. Isobutyl gallate exhibited no cytotoxic activity against Huh 7 and possessed strong activity (IC_50_ = 4.45 *μ*g/mL against DENV [177]. Gallic acid, fisetin, quercetin, and catechin inhibited infectious viral particles production against DENV-2 infected Vero cells [178–180]. Gedunin was evaluated against the DENV infected BHK-15 cells. Gedunin showed a significant reduction (EC_50_ = 10 *μ*M). In addition, the molecular docking study showed the strong interaction of the compound with the ATP/ADP binding site of the host protein (Hsp90) [181].

The study of thrombocytopenia (≤30,000/*μ*l) in adult dengue infections, leaves of *Carica papaya* extract, was enhanced platelet counts, TNF*α*, and IFN*γ* levels while it reduced IL-6 levels in patients [182]. In another pilot study was done in Srilanka, two doses of leaves extract at 8 h of intervals were found an increase in total WBC and platelet count within 24 h of treatment [183]. The 25 mL leave juice of *C. papaya* (two times a day for 5 days) was found an increase in total WBC and platelet count after 2 days of drug administration in Pakistani patients [124].

The aqueous leave extract of *C. papaya* was tested against DENV-infected THP-1 cells and its role on platelet augmentation. The leave extract was facilitated in platelet augmentation and showed antidengue activity by a significant reduction in the envelope expression, erythrocyte damage, nonstructural (NS1) proteins, lipid peroxidation, and intracellular viral load. In addition, thrombocytopenic rats administered with aqueous extract exhibited increased IL-6 and thrombopoietin levels [184]. In Indonesia, hydroalcoholic leave extract of *C. papaya* was given in capsule form to the patient having a thrombocyte count of <150,000/*μ*L along with 20% hematocrit. The recovery of patients was found faster via speedy increase platelets levels [185].

The leave extract of *Hippophae rhamnoides* was tested in DENV-2 infected BHK-21 cells. The extract was found potential antidengue activity by sustaining the cell viability in infected cells, decreasing TNF-*α* and increasing IFN-*γ* levels [186]. Aqueous extract of the *Scutellaria baicalensis* roots was tested against DENV 1-4 serotype-infected Vero cells. The extract exhibited strong virus replication property (IC_50_ = 74.33 to 95.83 *μ*g/mL for DENV 1-4 serotypes) [187]. The aqueous extract of *Solanum villosum* green berries showed the highest mortality against *Stegomyia aegypti* [154]. The silver nanoparticles (AgNPs) were synthesized from Holarrhena antidysenterica bark extract which showed strong larvicidal activity (LC_50_ = 9.3 ppm) as compared with other organic solvents and aqueous extract alone [188]. The synthesized AgNPs from Gmelina asiatica leave extracts showed potent larvicidal activity (LC_50_ = 22.44 *μ*g/mL) against Anopheles stephensi larvae [189].

### 4.3. Chemicobiological Data

Isolated compounds of medicinal plants and/or their derivatives may be one of the potential tools for the treatment of DENV and act against the vectors [190], such as essential oils [191], polyphenols [192], flavonoids [140], alkaloids [193], glycosides [194], and tannins [195].

**Figure 6:**
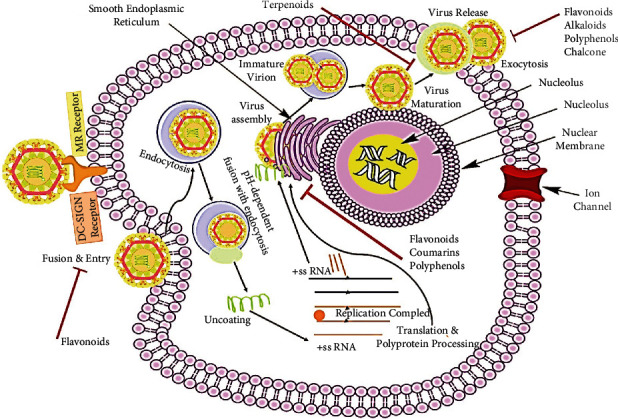
Potential antiviral mechanism and molecular targets of the bioactive compounds inhibiting viral entry and replication of dengue virus.

The antiviral mechanism of natural compounds inhibiting viral entry and replication is shown in Figure 6.

Infection of virus involves various stages:

- In the initial steps, DENV binds to cell receptors including mannose-binding receptor (MR) and DC-SIGN (dendritic cell-specific ICAM3 grabbing nonintegrin) receptor present at the surface of the cell, followed by fusion and entry
- Clathrin-mediated endocytosis and transport of DENV take place along with pH-dependent fusion with endocytosis
- The genomic ssRNA (positive-sense) is translated hooked on a polyprotein, which is smitten into all proteins
- Transcription and ribonucleic acid (RNA) replication occurs at the endoplasmic reticulum (ER) surface
- A synthesized dsRNA genomic virus is taking place at ER. At the ER, virions bud and are passaged to the Golgi, where DENV prM (membrane) protein is cleaved, and virion maturation takes place and is released by exocytosis

Natural compounds inhibit several proteins involved in the transcription as well as translation machinery essential in the DENV life cycle.

**Figure 7:**
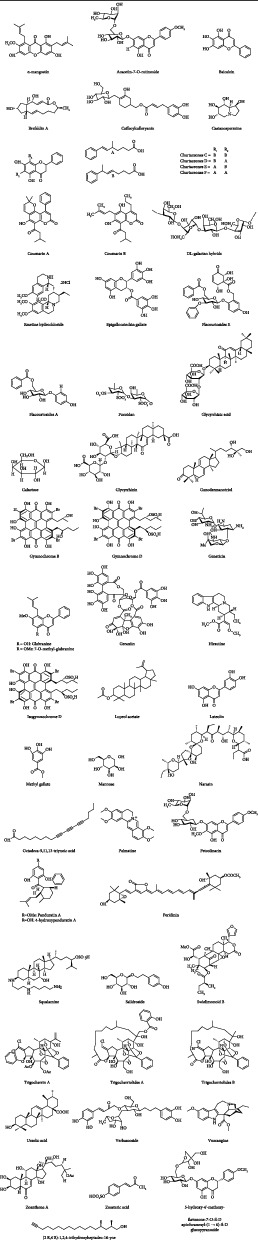
Chemical structures of natural compounds acting against dengue.

Furthermore, natural compounds block the virus replication by modulating the inflammatory redox-sensitive pathways and host cell signaling. Details of plant-derived natural compounds and their antidengue activities are stipulated in Table 2, and their chemical structures have been displayed in Figure 7.

Monocyte macrophages are thought to be the principal target cells for the DENV, the cause of dengue fever and hemorrhagic fever. Besides Ca^2+^, depletion of Mg^2+^ is also evident during binding of DENV to monocyte macrophages and cells of T cell and B cell lineages in in vitro studies [8]. It has been seen that the monocyte-derived macrophages discriminated in the presence of vitamin D3 restrict DENV infection and moderate the classical inflammatory cytokine (e.g., TNF-*α* and IL-1*β*, -4, and -10) response, where a reduced surface expression of C-type lectins, including the mannose receptor [214].

In another report, 1,25(OH)2D3 is evident to suppress the levels of IL-4 and IL-17A and modulate the levels of IL-12p70 and IL-10 in DENV infected U937-DC-SIGN cells and THP-1 macrophages, suggesting an immunomodulatory power that can ameliorate inflammation during dengue infections [196]. These findings have also complied with an earlier report [217]. In a clinical study, patients (*n* = 64), received a single dose of 200,000 IU vitamin D, was found to decrease the risk of dengue fever [220]. A challenge test done with 10 healthy individuals supplemented with 1000 or 4000 international units (IU)/day of vitamin D during 10 days suggested that 4000 IU/day of vitamin D represents an adequate dose to control DENV progression and replication [222].

## 5. Conclusions and Perspectives

To date, it is not possible to recognize intricate details and the complexity of the target of DENV of the other suitable vectors/secondary or hosts for its entrance, production, transmission, and pathogenesis. Several preventive measures have been taken; however, still, there is a deficiency of operative treatment modalities of DENV infections in human and pet animals. DENV-mediated imbalance of micronutrients may be one of the effective significances of numerous pathophysiological situations, such as Ca^+2^ depletion for muscle pain, irregular heartbeats, muscle weakness, fatigue, painful signs and symptoms, and deficiency of vitamin D in case of inflammatory conditions. The deficiency of vitamin D leads to oxidative stress and the inflammatory process [235], which may contribute to lowering the standard levels of platelet in patients with DENV. The internal bleeding and fatigue of the infected patients perform the leading functions for platelet deficiency.

Taken together, the biochemical markers and immunological parameters can be considered as important diagnostic tools in DENV infection. Besides preventive measures, the medicinal plants and their derivatives might be alternative tools in the treatment of DENV infection. Calcium along with vitamin D supplements can be used in DENV infection. More researches are necessary regarding to the successful diagnosis, prevention, and control of dengue worldwide.

## Figures and Tables

**Table 1:** Laboratory diagnostic approaches for DENV infection detection.

Clinical sample	Diagnostic approach	Methodology
Acute serum (1-5 days of DF) and necropsy tissue	Virus isolation	Mosquito or mosquito cell culture inoculation
	Nucleic acid detection	RT-PCR, real-time PCR
	Antigen detection	NS1 Ag rapid test, NS1 Ag capture ELISA, immunohistochemistry

Paired sera S1: acute serul from 1 to 5 days S2: convalescent serum 15-21 days	IgG or IgM seroconversion (S1 to S2)	ELISA
		HI
		Plaque reduction neutralization test

Serum after day 5 of DF	IgM detection	MAC-ELISA, IgM rapid tests (lateral flow)
	IgG detection	IgG ELISA, HI, IgG rapid tests (lateral flow)

Abbreviations: Ag: antigen; DF: dengue fever; ELISA: enzyme-linked immunosorbent assay; HI: hemagglutination inhibition assay; IgG: immunoglobulin G; IgM: immunoglobulin M; MAC: immunoglobulin M antibody capture; NS1: non-structural protein 1; RT-PCR: reverse-transcription polymerase chain reaction.

**Table 2:** Antidengue activities of natural compounds.

Botanical name	Plants part	Isolated compounds	Model	Results	References
*Garcinia mangostana*	Fruits	*α*-Mangostin	DENV infection in human peripheral blood mononuclear cells (PBMC) in vitro	↓ virus replication, ↓ TNF-*α*, ↓ IFN-*γ*, ↓ IL-6, ↓ MIP-1*β*, ↓ IP-10	[196, 197]
*Anacolosa pervilleana*	Leaves	Octadeca-9,11,13-triynoic acid	DENV NS_5_ RNA-dependent RNA polymerase (RdRp) assay in vitro	IC_50_ = 3 *μ*M	[198]
*Streptomyces aureofaciens*	Fermentation	Narasin	DENV2-infected hepatocytes Huh-7 cells in vitro	IC_50_ = 1 *μ*M ↓ viral protein synthesis	[199]
*Glycyrrhiza glabra*	Root	Glycyrrhizin	DENV serotypes1-3 in vitro	EC_50_ = 450, 174.2, 632.7 *μ*g/mL	[200]
		Glycyrrhizic acid	DENV2 infected Vero E6 cells in vitro	IC_50_ = 8.1 *μ*M	[201]
*Squalus acanthias*	Liver	Squalamine	Human endothelial cells HMEC-1 *in vitro*	↓ viral infection IC_50_ = 100 *μ*g/mL	[202]
*Zastera marina. Rees*	Marine eelgrass	Zoasteric acid	DENV serotypes (1–4) in vitro	IC_50_ = 24, 46, 14, 47 *μ*mol/L	[137]
*Quercus lusitanica*	Galls	Methyl gallate	DENV-2 infected C6/36 cells *in vitro*	↓ DENV-2 NS2B/3 protease IC_50_ = 0.3 mg/mL ↓NS1 protein	[203]
*Flacourtia ramontchi*	Stem bark	Flacourtosides A, E	DENV NS_5_ polymerase RdRp in vitro	IC_50_ = 9.3 − 9.5 *μ*mol/L	[204]
*Gymnochrinus richeri*	Stalked fossil crinoïd	Gymnochrome B	DENV-2, DENV-4 infected PS cells in vitro	ED_50_ = 0.029 nmol/mL	[205]
*Gymnochrinus richeri*	Living fossil crinoid	Gymnochrome D, isogymnochrome D	DENV-1 infected PS cells in vitro	Reduction of foci was smaller than 1 *μ*g/mL	[206]
*Arrabidaea pulchra*	Leaves	Verbascoside, caffeoylcalleryanin, ursolic acid	DENV-2 infected Vero cells in vitro	EC_50_ = 3.2, 2.8, 3.4 *μ*g/mL	[207]
*Trigonostemon cherrieri*	Bark and wood	Trigocherrin A, trigocherriolides A and B	DENV NS_5_ polymerase RdRp in vitro	IC_50_ = 12.7, 3.1, 16.0 *μ*mol/L	[208]
*Micromonospora rhodorangea*	Whole part	Geneticin	DENV-2 infected BHK cells in vitro	EC_50_ = 2 *μ*g/mL	[209]
*Castanospermum australe*	Seeds	Castanospermine	DENV-2 infection of Huh-7 and BHK-21 cells 10^5^ PFU of mouse-adapted DENV-2 in vitro/in vivo	IC_50_ = 1 *μ*M ↓ mortality in a mouse model Dose = 10, 50, and 250 mg/kg	[210]
*Coptis chinensis Franch*	Rhizomes	Palmatine	DENV-2 infected Vero cells in vitro	EC_50_ = 26.4 *μ*mol/L	[211]
*Psychotria Ipecacuanha*	Roots	Emetine hydrochloride	DENV-2 infected Huh-7, BHK-21 in vitro	IC_50_ = 0.5 *μ*M	[212]
*Distictella elongate (Vahl) Urb*	Leaves and fruits	Petcolinarin and acacetin-7-O-Rutinoside	DENV-2 infected Vero, LLCMK2 cells in vitro	EC_50_ = 86.4 and 11.1 *μ*g/mL	[213, 214]
*Scutellaria baicalensis*	Roots	Baicalein	DENV-2 infected Vero cells in vitro	IC_50_ = 6.46 *μ*g/mL	[180, 215]
*Cryptocarya chartacea*	Barks	Chartaceones C-F	Dengue virus NS_5_ RdRp inhibition in vitro	IC_50_ = 1.8 to 4.2 *μ*M	[216, 217]
*Boesenbergia rotunda* (L.)	Rhizomes	Panduratin A 4-hydroxypanduratin B	DENV-2 NS2B/NS3 protease in vitro	Ki (inhibitory constants) = 21, 25 *μ*mol/L	[123]
*Tephrosia s.p.*	Aerial parts	Glabranine 7-O-methyl-glabranine	DENV-2 serotype in vitro	70% inhibition IC_50_ = 25 mM	[134]
*Mimosa scabrella*	Seeds	Mannose/galactose (1 : 1)	DENV-1 (Hawaii strain) virus in vitro	↓ virus titer IC_50_ = 347 mg/L	[114, 131]
*Leucaena leucocephala*		Mannose/galactose (1 : 4)		↓ virus titer IC_50_ = 37 mg/L	[114, 131]
*Gymnogongrus torulosus*	Red seaweed	DL-galactan hybrids	DENV-2 serotype infected Vero cells in vitro	IC_50_ = 0.19 − 1.7 *μ*g/mL	[128]
*G. griffithsiae and Cryptonemia crenulata*		Sulfated *G3d* and *C2S-3* polysaccharides		IC_50_ = 1 *μ*g/mL	[125]
*Cladosiphon okamuranus*	Brown seaweeds	Fucoidan	DENV-2 infected BHK-21 cells in vitro	IC_50_ = 4.7 *μ*g/mL	[78, 218]
*Nephelium lappaceum L.*	Whole plant	Geraniin	DENV-2 E domain III (rE-DIII) protein in vitro	IC_50_ = 1.75 *μ*M	[219, 220]
*Scutellaria baicalensis*	Radices	Baicalin	DENV-2 (NGC strain) infected Vero cells in vitro	IC_50_ = 13.5 ± 0.08 *μ*g/mL	[221, 222]
*Camellia sinensis*	Dried leaves	Epigallocatechin gallate	Dengue virus (serotypes 1–4) infected Vero cells in vitro	EC_50_ = 14.8, 18, 11.2, and 13.6 *μ*M	[223]
*Zoanthus spp.*	Animal materials	Zoanthone A	DENV-2 NS_5_ polymerase in vitro	EC_50_ = 19.61 ± 2.46 *μ*M	[224]
*Mammea americana*	Seeds	Coumarin A Coumarin B	DENV-2/NG strain in vitro	EC_50_ = 9.6 and 2.6 *μ*g/mL	[225]
*Tabernaemontana cymosa*	Seeds	Lupeol acetate Voacangine		EC_50_ = 37.5 and 10.1 *μ*g/mL	[225]
*Angelica keiskei*	Roots	Brefeldin A	DENV serotypes (1–4) in vitro	IC_50_ = 54.6 ± 0.9 nM (DENV-2) IC_50_ = 61.32 ± 13.5, 57.9 ± 0.1, and 65.7 ± 6.3 nM (DENV − 1, 3, 4)	[226]
*Uncaria rhynchophylla*	Leaves	Hirsutine	DENV-1 infected A549 cells in vitro	EC_50_ = 1.97 *μ*M	[227]
*Viola yedoensis Makino*	Aerial parts	Luteolin	DENV infected HEK-293 T, A549, and BHK-21 cells in vitro	EC_50_ = 4.36 to 39.16 mM	[228]
*Persea americana*	Fruits	(2 R,4 R)-1,2,4-Trihydroxyheptadec-16-yne	DENV serotypes (1–4) in vitro	EC_50_ = 14.61, 10.98, 12.87, and 14.61 *μ*M	[229]
*Nephelium lappaceum*	Rind	Geraniin	DENV-2 RNA synthesis in Vero cells in vitro	IC_50_ = 1.78 *μ*M	[195]
*Palythoa mutuki*	Formosan zoanthid	Peridinin	DENV NS2B/NS3 protease in vitro	IC_50_ = 4.50 ± 0.46 *μ*g/mL	[230]
*Ganoderma lucidum*	Fruiting bodies	Ganodermanotriol		IC_50_ = 50, 25 *μ*M	[231]
*Faramea bahiensis*	Leaves	5-Hydroxy-4′-methoxy-flavanone-7-*O*-ß-d-apiofuranosyl-(1 → 6)-*β*-d-glucopyranoside	DENV-2 in HepG2 cells in vitro	↓ viral replication ↓ infected cell number	[232]
*Rhodiola rosea*	Roots	Salidroside	DENV serotype-2 infection in vitro	↓ DENV envelope protein ↑ RNA helicases	[233]
*Swietenia macrophylla*	Seeds	Swielimonoid B		EC_50_ = 7.2 ± 1.33 *μ*M	[234]

## Data Availability

The data used to support the findings of this study are available from the corresponding author upon request.
